# Hierarchical network modeling with multidimensional information for aquatic safety management in the cold chain

**DOI:** 10.1002/fsn3.613

**Published:** 2018-03-15

**Authors:** Lu Liu, Xinlei Liu, Wubin Li

**Affiliations:** ^1^ School of Transportation Ludong University Yantai China

**Keywords:** aquatic product, cold chain, hierarchical network, multidimensional information, quality safety

## Abstract

The cold‐chain information has characterized by the loss and dispersion according to the different collecting methods. The description for the quality decay factors of aquatic products can be defined as the multidimensional information. A series of nodes with multidimensional information are assembled to be hierarchies aiming at describing the environment conditions and locations in the supply chain. Each of the single hierarchy levels constitutes a sequence of node information in a network, which is applied as internal information analysis. The cross‐layer information structure is defined as “bridge” information which is able to record the information transmissions among every hierarchy from the point of view of the whole chain. The study has established a novel structured modeling to describe the cold chain of aquatic products based on a network‐hierarchy framework. An organized and sustainable transmission process can be built and recorded by the multidimensional attributes for the whole course of cold chain of aquatic products. In addition, seamless connections among every hierarchy are attainable by the environmental information records continuously to monitor the quality of aquatic products. The quality assessments and shelf life predictions are estimated properly as the risk control in order to monitor and trace the safety of aquatic products under the supply chain perspective.

## 1 INTRODUCTION

As a typical perishable food, the aquatic products with the abundant nutrition and high moisture are liable to suffer spoilage for the growth and reproduction of microorganisms and bacteria (Aung & Chang, 2014; Song & Ko, 2016). Biological hazards are the main factors leading to the deterioration of aquatic products. Temperature is the most critical factor affecting the rate of quality decay (Kim, Kim, Kim, & Jung, 2016; Wang, Ting, & Ip, 2013; Zhu, Ma, Yang, Xiao, & Xiong, 2016). Therefore, low temperature is conducive to control the quality decay and ensure the safety of aquatic products by inhibiting or delaying the growth of microorganisms and enzymes (Chen, Wang, & Jan, 2014; Laguerre, Hoang, & Flick, 2013; Le Grandois et al., 2013). At the same time, the cold‐chain management can reduce the quality decay rate of aquatic products effectively to prevent from biological hazards.

In the cold chain, people use artificial refrigeration technology to ensure the excellent quality of the aquatic products and reduce the loss. In the circulation of logistics, the most common technology is low temperature control which keeps the aquatic products in a specific low‐temperature environment thoroughly (Jun Yue, Liu, Li, & Zetian, 2013; Nissen & Zammit, 2011; Pramanik, Jana, Mondal, & Maiti, 2015; Shih & Wang, 2016; Tang, Piera, & Guasch, 2016).

In the whole process of cold‐chain production, the quality of the aquatic products attenuates rapidly and continuously. So the organized and sustainable information transmission is quite crucial for safety analysis of aquatic products throughout the cold chain (Heising, Dekker, Bartels, & van Boekel, 2012, Hoang, Flick, Derens, Alvarez, & Laguerre, 2012; Hoang, Laguerre, Moureh, & Flick, 2012; Laguerre, Duret, Hoang, Guillier, & Flick, 2015). With the organized full information records and integrated internal data in hierarchies, the seamless information transmissions across hierarchies are considered as a critical criterion for risk managements and quality safety assessments. Presently, there are several information collection and transmission problems as follows: information losses caused by insufficient correlation analysis and integration owing to the lack of full information records in the cold chain; and information fragments caused by inappropriate and inefficient data transmission among suppliers in the cold chain (Parreño‐Marchante, Alvarez‐Melcon, Trebar, & Filippin, 2014; Zanoni & Zavanella, 2012; Zhu, Zhang, Chu, He, & Li, 2014). According to the above problems, the cold chain is vulnerable with those hazards and risks, especially in the matter of quality safety analysis and shelf life estimation of aquatic products.

The information collection of the cold chain requires extremely strict organization in various aspects: the circulation information, such as producing, processing, storage, and distribution, and the additional information, such as time–temperature data, operating procedures, and environmental media conditions (Tsironi, Giannoglou, Platakou, & Taoukis, 2016; Wang et al., 2015). Environmental information is significant in product quality analysis as its major influence on the quality of aquatic products. As a matter of fact, the quality decay occurs continuously and irreversibly in the cold chain. As the integrated full records of information are required, the particular environmental information collection is necessary to be analyzed to accomplish accurate quality assessments (García et al., 2017; Long, 2016; Pasandideh, Niaki, & Asadi, 2015; Trebar, Lotrič, & Fonda, 2015; Xiao, He, Zetian, Mark, & Zhang, 2016). In summary, the purpose of the environmental information collection is to obtain intensive environmental information and the full processing records. In the process of information collection in the cold chain, quality assessments are implemented analytically with operating information and environmental information. Adapting to the information collection framework, the integrated records consisting of operating information, environmental information, and quality information are applied to estimate product quality precisely for the validity and safety of the cold chain. The complete information collection methodology is a critical reference for the shelf life prediction and the quality‐tracing procedures, which is considered as a real‐time safety monitoring and risk warning.

In this study, the quality decay factors of aquatic products are analyzed. By constructing multidimensional basic information nodes and embedding them into multilayer network structure, the composition of aquatic cold chain is divided into “dot” to “surface,” and a multilayer net “three‐dimensional structure” is built to collect and record the whole process of the cold chain of aquatic products. The general description of the cold chain is a network modeling based on node information in processing transmission. Aiming at internal information in the network, the integrated information hierarchies constitute orderly network with all of node information which is a description of full processed information transmission. A novel structure description of aquatic products is developed gradually to be a hierarchical network modeling of information description. Eventually, this modeling takes effects on comprehensive assessments and predictions in validity and safety of the cold chain.

## 2 RELATED WORK

In the most of related researches, the information modeling is studied in a view of information managements including information collection, information transmission, and information processing. There are some well‐known methodologies as follows: network management modeling, information record methodology with the workflow diagram and the Petri net, and multidimensional information description modeling. Data cube and OLAP data warehouse are commonly applied to describe node information of one kind of aquatic products as multidimensional information (Cui & Wang, 2016; Huang et al., 2015; Imani & Ghassemian, 2016, Lughofer & Sayed‐Mouchaweh, 2015; Rice, 2016; Uzam, Gelen, & Saleh, 2017; Wang, Zeshui, Fujita, & Liu, 2016). Wang, Li, Luo, and Fujita (2016) focused on the dynamic RSA for the multidimensional variation of an ordered information system and proposed a novel incremental simplified algorithm. Sifer and Potter (2013) combined distributional and correlational views of hierarchical multidimensional data to explore the data distribution and correlation. Ang and Wang (2015) used energy data with multiple attributes to analyze the changes in energy consumption over time. Kołacz and Grzegorzewski (2016) proposed an axiomatic definition of a dispersion measure that could be applied for any finite sample of k‐dimensional real observations. Boulila, Le Bera, Bimonteb, Gracc, and Cernessond (2014) presented the application of data warehouse (DW) and online analytical processing (OLAP) technologies to the field of water quality assessment. Santos, Castro, and Velasco (2016) presented the automation of the mapping between XBRL and the multidimensional data model and included a formalization of the validation rules. Data cube and OLAP data warehouse are the most applied to describe node information of cold chain of a single aquatic product as multidimensional information. Usman, Pears, and Fong (2013) presented a novel methodology for the discovery of cubes of interest in large multidimensional datasets. Kaya and Alhajj (2014) proposed and developed three different academic networks with a novel data cube‐based modeling method. Kapelko and Kranakis (2016) considered n sensors placed randomly and independently with the uniform distribution in a d‐dimensional unit cube. Julien Aligon, Gallinucci, Golfarelli, Marcel, and Rizzi (2015) proposed a recommendation approach stemming from collaborative filtering for multidimensional cubes. Blanco, de Guzmán, Fernández‐Medina, and Trujillo (2015) defined a model‐driven approach for developing a secure DW repository by following a relational approach based on the multidimensional modeling. Do (2014) applied online analytical processing (OLAP) to a product data management (PDM) database to evaluate the performance of in‐progress product development. Svetlana Mansmann, Rehman, Weiler, and Scholl (2014) introduced a data enrichment layer responsible for detecting new structural elements in the data using data mining and other techniques. Dehne, Kong, Rau‐Chaplin, Zaboli, and Zhou (2015) introduced CR‐OLAP, a scalable cloud‐based real‐time OLAP system, based on a new distributed index structure for the distributed PDCR tree. Network modeling is mainly a topological structure to describe information constitution and transmission (Sookhak, Gani, Khan, & Buyya, 2017). The links of information description are connected tightly and clearly. Demirci, Yardimci, Muge Sayit, Tunali, and Bulut (2017) proposed a novel overlay architecture for constructing hierarchical and scalable clustering of peer‐to‐peer (P2P) networks. Alam, Dobbie, and Rehman (2015) used a hierarchical agglomerative manner with HPSO clustering by execution time to measure the performance of our proposed techniques. Wang, Yang and Bin (2016) advanced a new hierarchical representation learning (HRL)‐based spatiotemporal data redundancy reduction approach.

The Petri net and workflow diagram are concurrent event records to describe computer processes (Cheng, Fan, Jia, & Zhang, 2013; Long & Zhang, 2014; Ribas et al., 2015; Wu, Wu, Zhang, & Olson, 2013). Application of the Petri network is one of the solutions in workflow diagram for its excellent feature of process records and information collection (Li, Wang, Zhao, & Liu, 2016; Nývlt, Haugen, & Ferkl, 2015; Zegordi & Davarzani, 2012). It is capable of recording and tracing information thoroughly. In particular, the ignition mechanism presents a clear connection among those pieces of information. Gamboa Quintanilla, Cardin, L'Anton, and Castagna (2016) presented a methodology to increase planning flexibility in service‐oriented manufacturing systems (SOHMS). Liu and Barkaoui (2016) surveyed the state‐of‐the‐art siphon theory of Petri nets including basic concepts, computation of siphons, controllability conditions, and deadlock control policies based on siphons. Vatani and Doustmohammadi (2015) proposed a new method of decomposition of first‐order hybrid Petri nets (FOHPNs) and introduced the hierarchical control of the subnets through a coordinator. Motallebi et al. defined parametric multisingular hybrid Petri nets (P‐MSHPNs), as a parametric extension of MSHPNs (Motallebi & Azgomi, 2015). Workflow diagram is a chart with proper symbols to indicate logical relationship of entire work records in a systematical organization and present the relationship between workflow connection and integrity as well as information flow sequence in cold chain. Ghafarian and Javadi (2015) proposed that workflow scheduling system partitions a workflow into subworkflows to minimize data dependencies among the subworkflows. Kranjc, Orač, Podpečan, Lavrač, and Robnik‐Šikonja (2017) presented a platform for distributed computing, developed using the latest software technologies and computing paradigms to enable big data mining based on a workflow. Liu, Fan, Wang, and Leon Zhao (2017) proposed a novel approach called data‐centric workflow model reuse (DWMR) framework to provide a solution to workflow model reuse. Park, Ahn, and Kim (2016) formalized a theoretical framework coping with discovery phase and analysis phase and conceived a series of formalisms and algorithms for representing, discovering, and analyzing the workflow‐supported social network. Hsieh and Lin (2014) applied PNML to develop context‐aware workflow systems using Petri net. Ribas et al. (2015) proposed a place/transition or Petri net‐based multicriteria decision‐making (MCDM) framework to assess a cloud service in comparison with a similar on‐premise service. The advantage of Petri Net is that the information can be classified with different colors according to the time coordinate records. However, by far, there is little research on the modeling methodology of the Petri network merged with workflow diagram for the information definition, information transmission, and supply chain structure analysis.

The descriptions and organizational structures are all practical, respectively. Multidimensional information is suitable at processing node information of aquatic products in detail. The Petri network and workflow diagram are applied for describing procedures of information transmission, while network modeling is built for hierarchical and structural descriptions. Currently, none of those methodologies is comprehensive enough for description and application. Furthermore, present research scopes are deficient in depth and width for studying the cold chain of aquatic products. The deficiency of information collection of the cold chain in depth, for instance, is lack of distribution nodes and location information, full‐dimensional environmental information, and processing information of aquatic products in the cold chain. Even worse, those types of inadequate information are not bounded up tightly and deeply. Meantime, the shortage of cold‐chain information records can cause data loss and fragmentation caused by few data interface adapters established between vendors during the process of information delivery. The information deficiencies mentioned above are considered as decisive and reliable approaches to quality safety assessments and validity estimation of the cold chain. In this research, the hierarchical network information modeling consisting multidimensional information is applied in the studies to deliver full‐dimensional and perspective description of aquatic products in cold chain.

## 3 CORRELATION ANALYSIS OF MULTIDIMENSIONAL INFORMATION

The multi‐attribute parameters are defined as a description of the same node in an omnidirectional vision. Then, the node information can be transmitted. The independent topological network structure is developed simultaneously. All of the nodes at the same stage in the cold chain are bound up to be a unit of hierarchical information. Even more, information transmission across different stages becomes independent hierarchical information. With this approach, the hierarchical network modeling can be established for acquiring complete full‐dimensional information based on multidimensional basic nodes.

**Figure 1:**
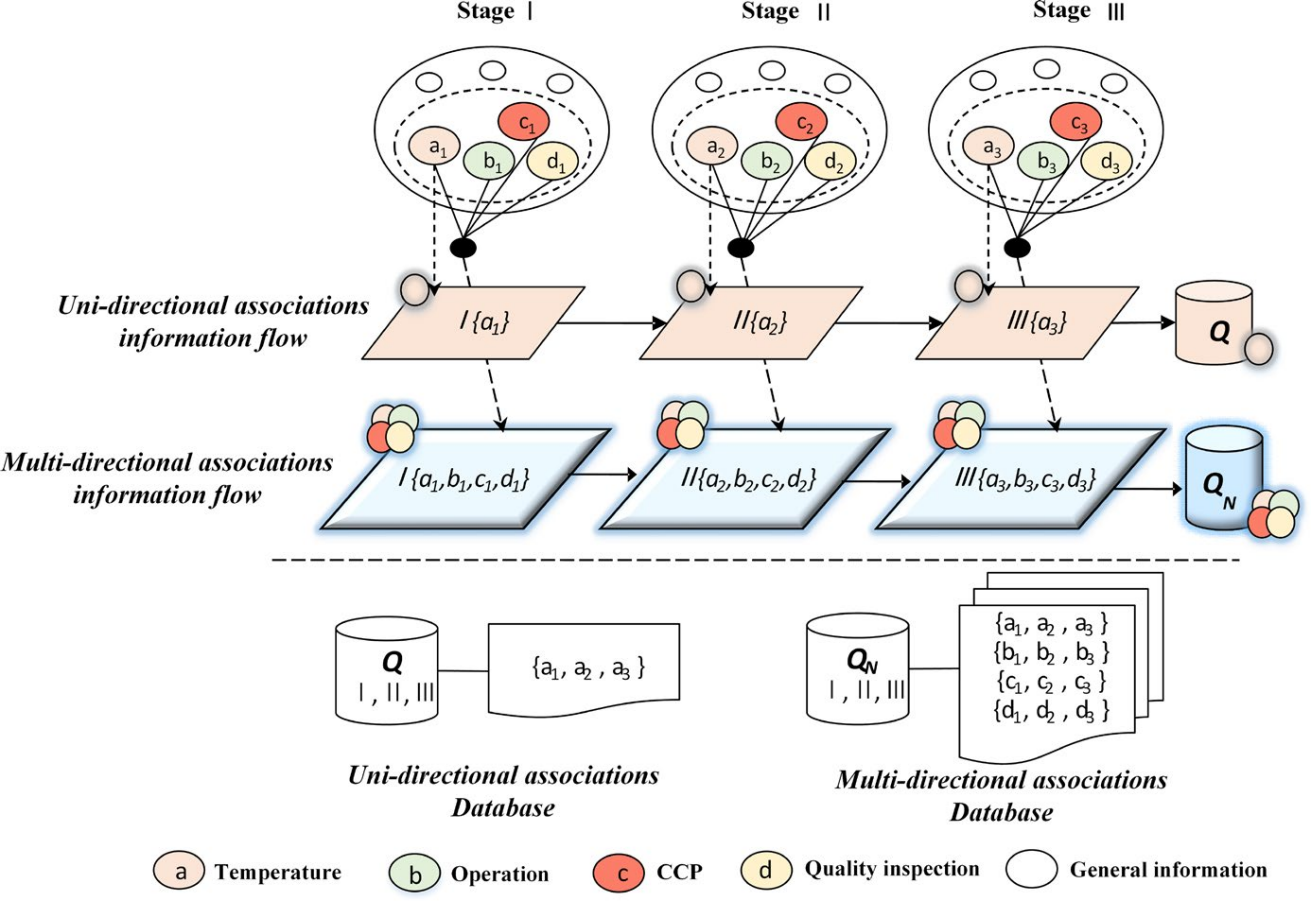
Multidimensional information architecture. Full‐dimensional information could be classified in multidimensional data to form a massive data analysis center as 3D information structure

According to time series and procedures of the cold chain, meta‐information is bound together tightly among points to set up netlike and hierarchical structure of 3D information connection and transmission on the basis of supply chain structure. The network information structure is composed of node information at the same stage. Plan‐metric network information is considered as a single unit of independent suppliers or “users” with the time series during the process of the cold chain. Network information located on the same layer is titled as a single hierarchy which is responsible for describing information of the same purchaser or unit. Technically, the hierarchical information is used to describe the same type of node information. Then, the new network information is generated through circulation at another stage. As it is illustrated in Figure 1, the comparison is made between multidimensional information structure and single information flow. The single information focuses on recording particular information of the supply chain, while the multidimensional data focuses on collecting and recording the multidimensional information of the supply chain. The comparison in a database is composed of two kinds of information flow records. Full‐dimensional information could be classified in multidimensional data to form a massive data analysis center. Applying to data mining algorithm, full‐dimensional information flow is recorded to implement safety analysis and risk warning during the entire cold chain of aquatic products.

By this method, the linkage and spreading structure of 3D hierarchical network information is developed in the pattern of recording and transmitting information to describe the whole information in the cold chain. In this research, an extensive information definition and modeling is applied for analyzing the cold chain based on 3D information transmission modeling exhaustively and completely.

### 3.1 The depth definition of aquatic product information in the cold chain

Full‐dimensional information structure is advanced in describing aquatic product information in the cold chain intensively. The multi‐attribute parameters are applied to collect and record the full‐dimensional information of the cold chain, such as node information of the supply chain, processing information, and environmental information.

Multi‐attribute parameters are defined by basic node structure of the cold chain only if basic information components are analyzed. In other words, there is a multi‐information description for even a single node of aquatic products. For example, one of the manufacturing processes is taken as a piece of node information, and its attributes are as follows: location, time, temperature, water quality, procedures, participant data, and so on. The nodes are transmitted along with its attribute parameters during circulation process of the cold chain consecutively and completely.

Definitely, through the use to this method, the overall analysis of the cold chain is accomplished. There are three categories of necessary information that should be recorded: (1) circulation node information of the supply chain including logistics node, location, and supplier information; (2) processing information of aquatic products including processing methods and procedures, processing facilities, operating time, and operators; and (3) environmental information including environmental media (air, liquidity, solidity—such as internal organs of fish), and key environmental information which influences quality decay significantly including time, environmental temperature, and moisture in a specific environmental media. Basic node information is indicated by multidimensional structure information consisting of those three categories. The multidimensional node information is featured by multi‐attribute parameters of aquatic products in the cold chain. Combining with three categories of basic information, multidimensional node information is established for the depth information description of the cold chain.

Through establishing a creative basic node of information transmission, the cold chain of aquatic products is analyzed and evaluated in the ground of multidimensional node information, such as nodes of the supply chain, specific manufacturing and processing information, and environmental information of aquatic products. In this way, intensive information of aquatic products is developed and perfected with the multidimensional basic nodes for full‐dimensional information description of the cold chain. Meta‐information is defined as basic node description which is the smallest unit of node information description in processing. Definition of full‐dimensional attributed node information is mainly relied on processing information of aquatic products. It is processing meta‐information based on time series or procedures with parameters to describe multidimensional information of the cold chain in all aspects.

### 3.2 The width definition of aquatic product information in the cold chain

In the view of extensive scope of aquatic product information in the cold chain, meta‐information is divided into two types: the internal hierarchical information and the bridging information. The node information in the internal hierarchy is applied as information description of one stage in the cold chain. A bunch of meta‐information on the same layer is assembled together to be a network structure for describing aquatic products, such as processing stage, storage stage, transportation stage, and distribution stage. Regarding multidimensional attributes, the internal meta‐information on each layer elaborates full‐dimensional information of aquatic products on an independent stage of the cold chain. Information structure of the stages is composed of a network structure with node information on the same stage to describe the information transmission of the supply chain.

From the perspective of supply chain, the cold chain of aquatic products was analyzed, and there was a kind of relatively special type of information. An interhierarchy information occurs in the circulation in different factories or by different suppliers. Thus, there are two types of interhierarchy information including location diversion and supplier diversion. Furthermore, it is the crucial link to connect the upper hierarchy and lower hierarchy. The upper hierarchical information only can be transmitted consecutively if interhierarchy information is connected seamlessly. Or else, deficient information of the cold chain is the definite consequence.

In this research, the link of node information is defined as “bridge” node information which connects each hierarchical partition and transmission of aquatic products in the cold chain. The “bridge” information is a combination of two types of node information connected from the ending node of the upper hierarchy to the beginning node of lower hierarchy. Each single hierarchy exists independently and orderly. Specifically, the definition of hierarchy can be variety of location diversion, supplier diversion, and logistics diversion. Additionally, special hierarchy information is defined based on node information attributes, such as periodical attributes or operator attributes, as the specific information analysis and assessments. The hierarchical 3D network information modeling is built up to connect network information on the same hierarchy and transmit information among every hierarchy through bridge nodes. This method is able to cover information recording of the cold chain extensively and completely.

**Figure 2:**
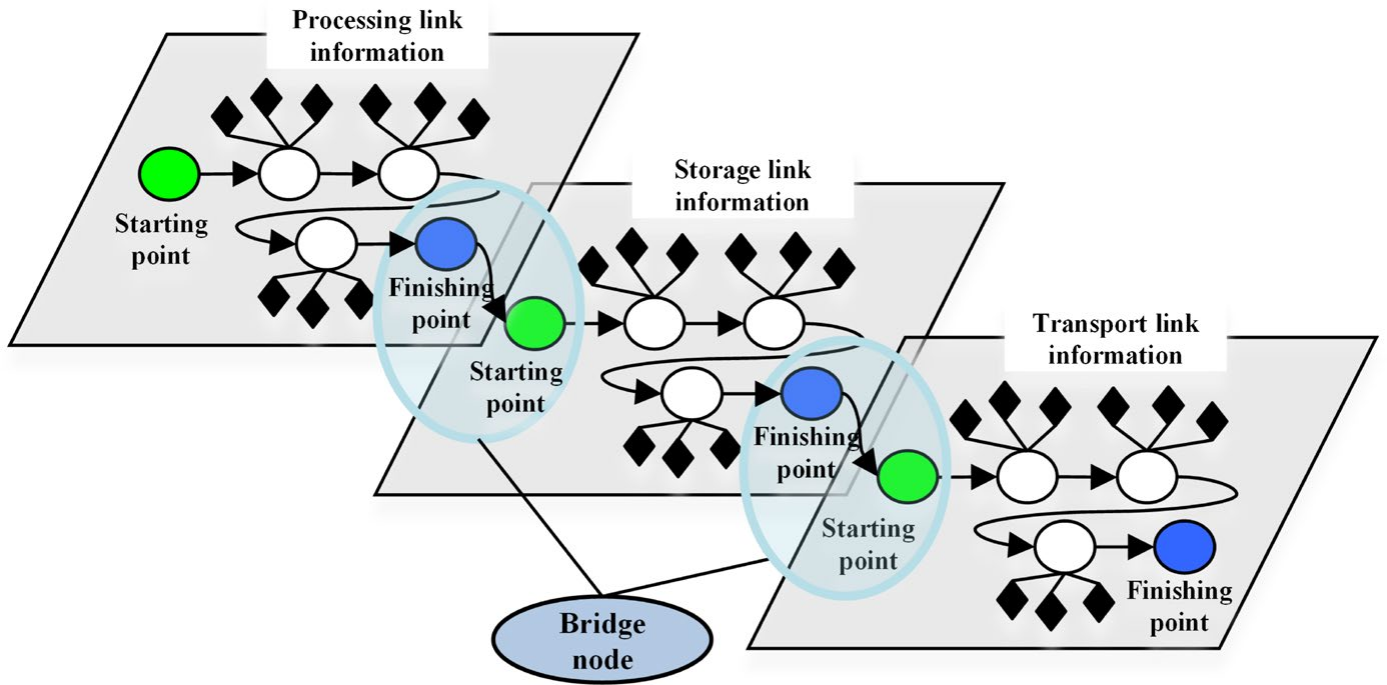
The bridge node information architecture. The network structure of the processing stage consists of all of processing information. The green node is the beginning of processing information, while the blue node is the ending of processing information. The bridge is just a connection between the ending of the processing stage and the beginning of the storage stage. Another bridge is a connection between the ending of the storage stage and the beginning of the freightage stage, and so on. By this way, information and data direction can be explained by the stage information and the connecting information

Figure 2 illustrates the processing stage, storage stage, and freightage stage of the cold chain. In the chart, network structure of the processing stage consists of all of processing information. The green node is the beginning of processing information, while the blue node is the ending of processing information. The bridge is just a connection between the ending of the processing stage and the beginning of the storage stage. Another bridge is a connection between the ending of the storage stage and the beginning of the freightage stage, and so on. By this way, information and data direction can be explained by the stage information and the connecting information. Table 1 shows a constructing parameter definition of the multidimensional information model.

**Table 1:** Parameter definitions of the multidimensional information model

Type of information	Definition	Parameter
Multi‐attributes/parameters	Multiparameters of meta‐information	$\forall p\in P:\left[Type\left(I\left(p\right)\right)=C{\left(p\right)}_{\mathrm{MS}}\right],I\in \left[P\to Exprs\right]$
Node	The smallest unit of processing as basic information description of aquatic products.	$M=\left\{m|m_{i}\in M,i=1,\cdots ,n\right\}$
Hierarchy information	Network structure of information transmission composed of adjoining nodes on the same hierarchy.	$\psi =\sum_{i=1}^{n}M_{i}$
Single linkage	Vector information for directing information transmission.	$\Phi \in \left[A\to Exprs\right]$, Φ the arc function
Bridge (double linkage)	Combination of connecting information including ending node on upper hierarchy, beginning node on lower hierarchy and reversible vector arc between nodes.	$\forall a\in A:\left[\begin{array}{c} Type(\Phi (a))=C(p(a))\wedge Type(Var(\Phi (a))) \end{array}\right]$ *A:* the bridge node set

### 3.3 The colored Petri network modeling

In the modern world, during the course of production in logistics, many of the modeling methodologies are applied, such as the control theory, Lagrange relaxation methodology, SCOR modeling methodology, and other advanced modeling methodologies. In this study, the structured model, especially like the colored Petri network, is more superior in risk control, validity analysis, and data tracking during the entire process of the cold chain. Compared with other modeling methodologies, the Petri network is more practical in analyzing structures and behaviors of the cold chain. Moreover, the colored Petri network is advanced enough to be inserted with multidimensional data, such as time, procedures, and any other environmental factors, which is exactly what we are working on.

The basic definition of the Petri network: The necessary and sufficient conditions of triple *N* = (*P*,* T*;* F*) structured network (net) are as follows:

1. *P*∩*T* = Φ presents the location and the variation which are totally two different elements;
2. *P*∪*T* ≠ Φ presents at least one element existing in the network;
3. *F* = (*P* × *T*) ∪ (*T *× *P*) presents unidirectional connection between the location and the variation and prevents any direct connections among the same type of elements.

The colored Petri network uses colorful symbols for more vivid illustrations. Sometimes, a set of colors is added or transferred. Even an I/O function is transferred for more specific explanation.

Σ=(P,T;F,C,I−,I+,M0)

For example:

(*P*,*T*;*F*) broadly oriented network is the base net of Σ.

*C* is a color set which includes the following:

As to *p* ∈ *P*,* C* (*p*) is the nonempty finite set located on *p* with or without colors.
As to *t* ∈ *T*,* C* (*t*) is the nonempty finite set transferred to *t* with or without colors.
*I*− and *I*+ are negative and positive functions for *P* × *T* and *T *× *P*
  ∀p∈P,t∈TI−(p,t)≠0∨I+(t,p)≠0
  ∀t∈T,p∈PI−(p,t)≠0∨I+(t,p)≠0
*M*_0_ is an initial symbol for Σ.

In the colored Petri network, different colors are presenting different multidimensional information, such as green nodes for processing information of aquatic products, purple nodes for circulation information of the cold chain, and blue nodes for environment information. Therefore, the multidimensional information fusion and risk information management of the cold chain have been accomplished ultimately.

## 4 DECISION‐MAKING MODEL BASED ON THE HIERARCHICAL NETWORK STRUCTURE

Based on the ergodic process of aquatic products, the multi‐attribute parameters are supervised by the thorough records, which is equipped with hierarchical network information modeling of the entire cold chain. The description of information transmission contributes to extract more complete information of aquatic products in storage during the cold chain. Various independent functions of analysis and assessments are implemented regarding the information extracted from the cold chain as follows: safety assessments, validity estimation, information tracking, real‐time monitoring, and risk warning of aquatic products in the cold chain.

In the matter of multi‐attribute parameters, the clustering of multidimensional information attributes are extracted from the parameter sets. According to the research, there are three types of clustering by extracting attributes of multidimensional information as follows: behavior information clustering, environmental information clustering, and quality information clustering. Behavior information clustering concentrates on extracting multidimensional node information containing operational attribute parameters. Briefly, a set of operating information description is interpreted as multidimensional attribute information which is extracted from the circulation in the cold chain with time series. So it can be applied to analyze the operating safety and the chain management of aquatic products. Furthermore, it is also capable of collecting information in real time. Environment information clustering concentrates on extracting multidimensional node information containing the full‐dimensional environmental attribute parameters. The set of environmental information description is associated with attributes of full‐dimensional environmental media, including temperature, moisture, and ergodic time, which is extracted from the circulation of the cold chain with time series. Therefore, the congregation of full‐dimensional environmental data is considered as a risk warning to quality safety of aquatic products and environmental monitoring in the cold chain. Quality information clustering is the integrated analysis on the ground of behavior transinformation and environmental transinformation.

The three types of clustering information are applied for the evaluation, management, and decision support of aquatic products in the cold chain. Safety conditions of the cold chain are analyzed on the basis of factors which have significant impacts on the quality safety of aquatic products. The conditional information is divided into two types: the safety conditions of operating protocols and the safety conditions of product quality in the cold chain. According to the decision‐making objects of operating protocols and quality assessments, the decision‐making of safety conditions is made based on information analysis extracted from the network hierarchical modeling.

The decision‐making objects taken as one of critical conditions are so decisive for the quality safety of aquatic products in the cold chain. The decision‐making process contains three elements including the decision‐making objects, methods, and results. Nevertheless, the three elements are distinguishable according to different decisions as it is illustrated in Table 2.

**Table 2:** Elements of safety decision of aquatic products in the cold chain

Decision targets	Decision objects	Decision methods	Decision results
Operation protocols	Time series of node in the cold chain	HACCP protocol	Time limits and protocols of each batch of processes in the cold chain
		GMP Protocol	
	Operation attribute parameters	T‐T‐T protocol	Process for each batch of aquatic products
Quality assessments	The attribute parameters by time series in the cold chain	Labuza quality assessment model	Quality change threshold of each batch of processes in the cold chain

Referring to the decision‐making modeling, the result is demonstrating the outputs of operating protocols or quality assessments of aquatic products. Decision‐making objects mean the conditions of operating safety and states of product quality for each batch of aquatic products in the circulation of the cold chain. Decision‐making process is conducted by the decision‐making methods. Besides, further discussion is made on the specific decision‐making process related to the operating procedures and the quality assessment of the product.

### 4.1 The evaluation of operating safety modeling

The evaluation of operating safety is defined as safety analysis and safety assessments of aquatic products during the processing practices in the cold chain from the view of operating managements. There are two main protocols commonly applied as follows: Hazard Analysis and Critical Control Point (HACCP) and Good Manufacturing Practice (GMP). In this research, regarding the criterion of qualified operating procedures and the limited time requirements, the operating safety is analyzed and evaluated by extracting the complete environmental information clustering and behavior information clustering from the hierarchical network modeling.

The operating instructions or any quick response strategies are executed without delay only if the decision‐making information is made based on information description of quality safety analysis which is deduced from the decision support modeling.

Based on the analysis of environmental temperature fluctuations, shelf life quality of aquatic products can be analyzed by the Baranyi model to evaluate aquatic product quality and analyze the overall safety level of cold‐chain aquatic products. This study mainly considers the influence of temperature on the shelf life quality of aquatic products.

The Baranyi model was used to fit the curve of bacterial growth and proliferation, and the square root was used to describe the effect of temperature on growth kinetics of bacteria. The model was improved to fit the growth status of microorganisms under fluctuating temperature. Temperature fluctuation experiment was designed for verification.

#### 4.1.1 Constant temperature

The Baranyi equation was used to fit the relationship between the number of microorganisms and time variation; thus, the initial bacterial colonies, period of delay, specific growth rate, and maximum bacterial concentration (Baranyi & Roberts, 1994; Hanhuang, 2013; Santillana Farakos, Frank, & Schaffner, 2013; Shamshad, Riaz, Zuberi, & Qadri, 1990) were obtained.

(1) $$N\left(t\right)=y_{0}+\mu_{\max}F\left(t\right)-\ln\left(1+\frac{e^{\mu_{\max}F\left(t\right)}-1}{e^{\left(N_{\max}-N_{0}\right)}}\right)$$

(2) $$F\left(t\right)=t+\frac{1}{v}\ln\left(e^{-vt}+e^{-h_{0}}-e^{{}_{{}^{{}^{\left(-vt-h{}_{0}\right)}}}}\right)$$

where *T*—storage time, d; *N*0—the initial number of microorganisms, l g CFU/g; *N*_max_—maximum value of the general number of microorganisms during storage, l g CFU/g; μ_max_—maximum specific growth rate of microorganisms, d−1; ν—h_0_, parameters, constants.

Level 2 model: The influence of temperature on growth state of microorganisms can be represented by Level 2 model (square root model) as follows:

(3) $$\sqrt{\mu_{\max}}=b\left(T-T_{\min}\right)$$

where μ_max_ = μ_0_; *b*, a constant, *T*_min_, the minimum temperature for the growth of microorganisms.

#### 4.1.2 Fluctuating temperature

Dynamic prediction of pseudomonas growth under fluctuating temperature:

When *t *= *d*_*t*1_ + *d*_*t*2_ + … + *d*_*ti*_,

(4) $$N\left(t_{i}\right)=y_{i-1}+\mu_{i}F\left(t_{i}\right)-\ln\left(1+\frac{e^{\mu_{i}F\left(t_{i}\right)}-1}{e^{\left(N_{\max}-N_{i-1}\right)}}\right)$$

(5) $$F\left(t_{i}\right)=t_{i}-t_{i-1}+\frac{1}{v}\ln\left(e^{-v\left(t_{i}-t_{i-1}\right)}+e^{-h_{0}}-e^{\left(-v\left(t_{i}-t_{i-1}\right)-h_{0}\right)}\right)$$

where *d*_*ti*_ (*i* = 1,2,3,…) is the short time interval under an assumed constant temperature and *N*(*ti*) is the bacterial number at *d*_*ti*_. *N*_0_ is the initial number of microorganisms when *t* = 0, and *N*_max_ is the maximum number of microorganisms when they increase to the steady period. The influence of temperature on the growth state of microorganisms can be represented by the square root model, that is, $\sqrt{\mu_{i}}=b\left(T-T_{\min}\right)$, where μ_*i*_ is the maximum specific growth rate at *d*_*ti*_. Using the above method, shelf life of aquatic products can be accurately estimated.

Regarding the above modelings, the quality assessments are provided as a consolidated foundation of analysis and definition for the quality safety of aquatic products under various temperatures in the cold chain. All of information at each stage is applied to confirm the fluctuated temperature environments, which is necessary to select one of the proper assessment modelings for analyses and evaluations of the quality safety. Those modelings are also supportive to make correct decisions and quick response strategies based on the quality safety states of aquatic products.

## 5 EXPERIMENTS AND RESULTS

**Figure 3:**
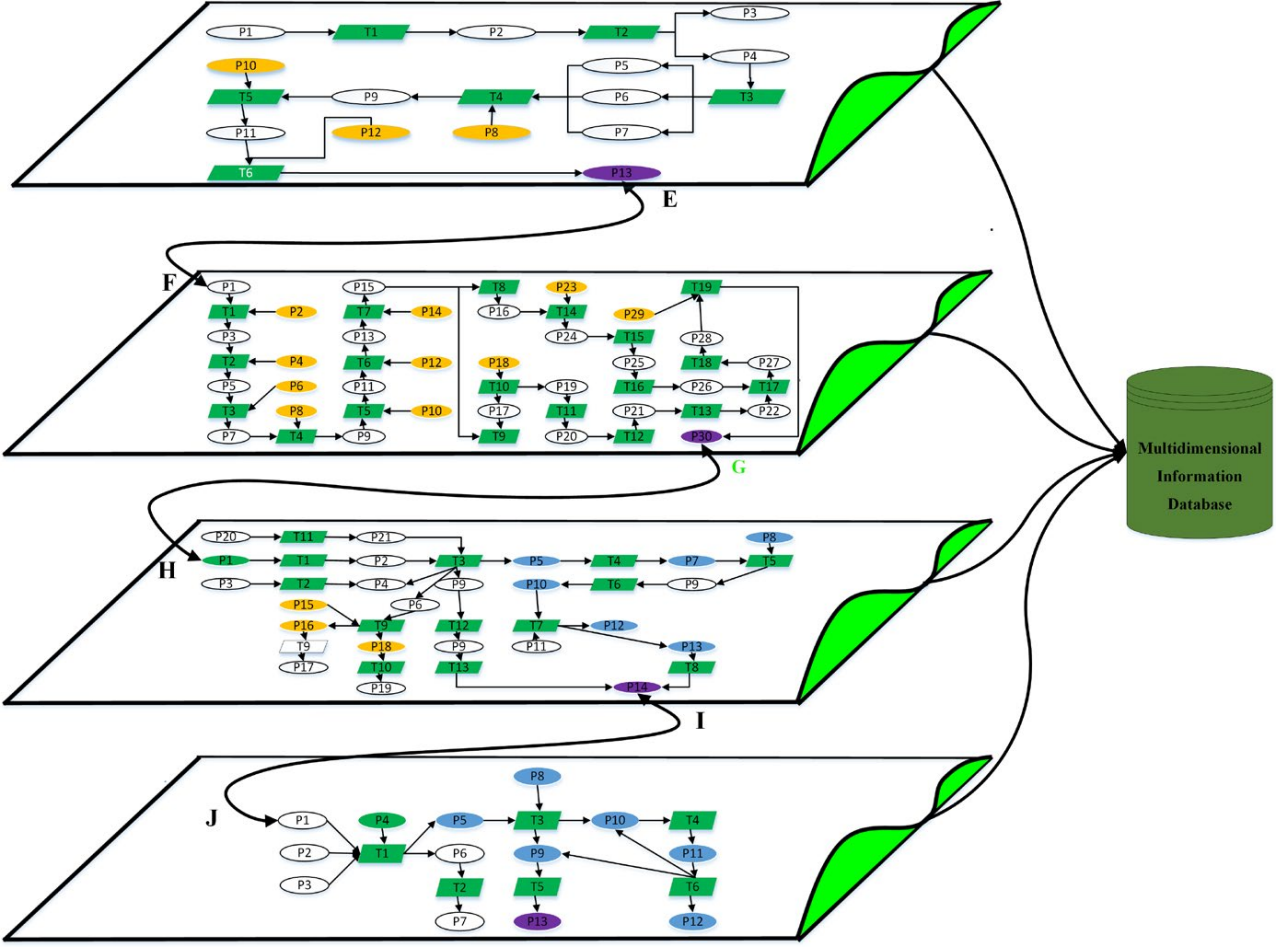
The multihierarchical network. In the 3D network‐hierarchy modeling, the circulation status of each node is elaborated through describing processing and environment information based on the attribute value of multidimensional nodes

In this research, the cold chain of tilapia is considered as an experimental object during cultivation, processing, storage, and transportation. Multidimensional node information of tilapia is involved in quality safety assessments and decision‐making strategies of risk warning in the cold chain. The Petri net, which is a simulation tool, is applied to establish a “network‐hierarchy” modeling for information transmission in the cold chain. In the 3D network‐hierarchy modeling, the circulation status of each node is elaborated through describing processing and environment information based on the attribute value of multidimensional nodes. Figure 3 indicates the process of information transmission at each of the independent stages of the cold chain.

Referring to the tilapia, there is a complex network structure on every hierarchy of modeling to describe the transmission process of multidimensional information including the cultivation stage, the processing stage, the storage stage, and the transportation stage. The nodes of bridges “E F G H I J” are defined as links between two separate layers to establish a 3D transmission structure of multihierarchical information. The impressive framework of the structure represents the transmission and behaviors powerfully. Meanwhile, the analysis of the cold chain is accomplished completely and efficiently. As it is shown in Figure 3, the green color of multidimensional node is used as the attribute information of temperature. The purple node is crucial parameter of inspection. The white represents circulation of products in the cold chain. The blue node is traffic information tagged by GPS. The information communion center is established as a platform to share information of every stage, such as the processing stage on the ship, the transportation stage, the processing stage on the shore, and the storage stage. At the processing stage on the ship, the nodes of bridges “EF” (*EP*_*13*_ *, FP*_*1*_), “GH” (*GP*_*21*_ *, HP*_*1*_), and “IJ” (*IP*_*12*_ *, JP*_*1*_), which are the crucial connections between the processing stage and the transportation stage, represent that tilapia have been delivered to the next stage. The nodes of bridge are considered as key nodes of information flows, which are extracted from the information communion center as it is required. Tangible information flows are consolidated to be a hierarchical modeling that the entire cold chain of aquatic products is visualized. Applying with this framework, all of data referring to the cold chain is stored in a multidimensional database.

Before entering the processing stage, information description of tilapia is shown in Table 3. Regarding the level of tilapia which have not been entering the processing stage, the information is defined by nodes in the Petri net from m_1_ to m_11_ with the multidimensional information description. The content of a token T is defined as *C*(*m*_*x*_:*m*_*y*_) for the description of information transmission mode to record the association with input and output nodes. The captured tilapia are ready to be processed as long as the temperature sensors are launched by the modeling. In this stage, three levels of tilapia are transferred to the main network with information flows. Therefore, the information tracing is available at the transparent processing stage with more details.

**Table 3:** Information description of tilapia before entering the processing stage

Place	Content	Place	Content	Token	Content
P1(m_1_)	Captured tilapia	P8(m_8_)	Time‐temperature data	T1	Deliver tilapia to the factory
P2(m_2_)	Delivered tilapia	P9(m_9_)	Tilapia being processed with CO	T2	Receiving inspection of raw materials
P3(m_3_)	Unqualified tilapia after being inspected	P10(m_10_)	Time‐temperature data	T3	Assorting tilapia according to different specifications
P4(m_4_)	Qualified tilapia after being inspected	P11(m_11_)	Bloodletting and disinfection of tilapia	T4	CO processing
P5(m_5_)	A class tilapia	P12(m_12_)	Time‐temperature data	T5	Bloodletting and disinfection
P6(m_6_)	B class tilapia	P13(m_13_)	Tilapia in the processing stage	T6	Delivering to the processing workshop
P7(m_7_)	C class tilapia				

At the processing stage, the information is shown in Table 4 and Table 5. The initial signs of places are P1, P2, P9, P13, P15, and P20, which are used to describe the multidimensional information m1, m2, m9, m13, m15, and m20 at the processing stage. The ending place of the transportation stage is the input place at this stage. The processing stage is triggered by the ignition. The information of related parameters in Table 5 is used for describing transmission information of tilapia at the processing stage. Through the Petri network simulation, the tokens from T1 to T4 are applied to indicate the transmitting relationship among the attributes of multidimensional information.

**Table 4:** Information description of the processing stage of tilapia

Place	Content	Place	Content	Place	Content
P1(m_1_)	Unloading tilapia	P11(m_11_)	Skin of tilapia being removed	P21(m_21_)	Weighting tilapia
P2(m_2_)	Time–temperature data	P12(m_12_)	Time–temperature data	P22(m_22_)	Tilapia in bulk
P3(m_3_)	Disinfected tilapia	P13(m_13_)	Classified tilapia	P23(m_23_)	Time–temperature data
P4(m_4_)	Time–temperature data	P14(m_14_)	Time–temperature data	P24(m_24_)	Vacuum packing tilapia
P5(m_5_)	Refrigerated tilapia	P15(m_15_)	Washing tilapia	P25(m_25_)	Refrigerated tilapia
P6(m_6_)	Time–temperature data	P16(m_16_)	Tilapia in individual packages	P26(m_26_)	Weighting tilapia
P7(m_7_)	Disinfected tilapia	P17(m_17_)	Tilapias in bulk	P27(m_27_)	Qualified tilapia after metal detection
P8(m_8_)	Time–temperature data	P18(m_18_)	Time–temperature data	P28(m_28_)	Packed tilapia
P9(m_9_)	Tilapia being processed through 3 necessary steps	P19(m_19_)	Refrigerated tilapia	P29(m_29_)	Time–temperature data
P10(m_10_)	Time–temperature data	P20(m_20_)	Ice‐glazed tilapia	P30(m_30_)	Tilapia in the storage stage

**Table 5:** Relationship description of tilapia at the processing stage

Token	Content	Token	Content
T1	Disinfection of raw material inlets	T11	Ice glazing
T2	Refrigeration with crushed ice	T12	Weighting
T3	Disinfection	T13	Packing in big plastic bag
T4	3 steps of processing tilapias (skin removing, scaling and guts removing)	T14	Vacuum packing
T5	Skin removing (shallow skin removing and deep skin removing)	T15	Tilapias refrigerated in the individual quick freezing machine
T6	Classification	T16	Weighting
T7	Washing and disinfecting process	T17	Metal detection
T8	Individual package	T18	Packing in cartons
T9	In bulk	T19	Being refrigerated in warehouse
T10	Tilapias refrigerated in the individual quick freezing machine		

The data of intensive temperature monitoring is uploaded to the processing center by sensors for different batches of tilapia (Table 6). Regulation is unnecessary when the temperature fluctuates within limit, which is considered as the normal temperature. Environmental temperature information is transmitted to the next stage when the aquatic products are delivered. During this period, time–temperature data is uploaded to the information communion center as a reference of quality assessments. Furthermore, barriers between each of the two stages are eliminated by visualizing the information of the cold chain at each of the stages.

**Table 6:** Information description of tilapia at the storage stage

Place	Content	Place	Content	Token	Content
P1(m_1_)	The first batch of tilapia	P8(m_8_)	Standard parameters of temperature fluctuations	T1	Refrigeration in warehouse
				$C\left(m_{1},m_{2},m_{3},m_{4}:m_{5},m_{6}\right)$	
P2(m_2_)	The second batch of tilapia	P9(m_9_)	Standardized temperature fluctuations	T2	Delivering from warehouse on order.
				$C\left(m_{6}:m_{7}\right)$	
P3(m_3_)	The third batch of tilapia	P10(m_10_)	Exceeding portion beyond the standard temperature fluctuations	T3	Temperature examination
				$C\left(m_{5},m_{8}:m_{9},m_{10}\right)$	
P4(m_4_)	Temperature sensors	P11(m_11_)	Regulated temperature	T4	Temperature regulation
				$C\left(m_{10}:m_{11}\right)$	
P5(m_5_)	Time‐temperature data	P12(m_12_)	Standard parameters of temperature fluctuations	T5	Uploading time‐temperature data
				$C\left(m_{9}:m_{13}\right)$	
P6(m_6_)	Tilapia at the refrigeration stage	P13(m_13_)	Information database of the cold chain	T6	Temperature examination
				$C\left(m_{11}:m_{9},m_{10},m_{12}\right)$	
P7(m_7_)	Tilapia delivered from warehouse				

As it is shown in Tables 7 and 8, P1 presents tilapia delivered at the transportation stage. After being inspected, unqualified tilapia are returned to the last stage for any possible urgent reprocessing. During the procedures, application of information flows involves time‐temperature information and operating information. The simulated procedures are imitating the entire process and conditions that the tilapia are experienced from the refrigeration trucks to the unloading stage. According to P12, there is always a token acting as an ignition during the circulation of the cold chain. Once the temperature decreases within the tolerant range, the circulation is terminated immediately. The numbers of place tokens are limited to be less than 3 which is available. P15 is the information records containing fluctuated environmental temperatures and the period of time when it is exceeding the tolerant range. Serving as a transitional part, establishment of the modeling is supplementary for data losses during the transportation in the cold chain. Eventually, the information for cold chain becomes integrity genuinely.

**Table 7:** Information description of tilapia at the transportation stage

Place	Content	Place	Content
P1(m_1_)	Temperature sensors	P13(m_13_)	Qualified parameters of the standard temperature fluctuation
P2(m_2_)	Await temperature sensors	P14(m_14_)	Information communion center of the cold chain
P3(m_3_)	Await delivered tilapia	P15(m_15_)	Quality parameters of inspection
P4(m_4_)	Await delivered tilapia	P16(m_16_)	Qualified quality
P5(m_5_)	Time‐temperature records	P17(m_17_)	Tilapia delivered to the next stage
P6(m_6_)	Delivered tilapia	P18(m_18_)	Unqualified quality
P6(m_6_)	Time‐temperature data	P19(m_19_)	Urgent processed tilapia
P7(m_7_)	Standard parameters the of temperature fluctuations	P20(m_20_)	GPS system
P8(m_8_)	Exceeding parameters of temperature fluctuations	P21(m_21_)	GPS system in holding stage
P9(m_9_)	Regulated temperature	P22(m_22_)	Locating conditions of GPS
P10(m_10_)	Standard parameters of the temperature fluctuations	P23(m_23_)	Uploading GPS data
P11(m_11_)	Exceeding parameters of temperature fluctuations		

**Table 8:** Relationship description of tilapia at the transportation stage

Token	Content	Token	Content
T1	Sensors are cleared and verified	T8	Uploading time‐temperature data
$C\left(m_{1}:m_{2}\right)$		$C\left(m_{13}:m_{14}\right)$	
T2	Truck loading	T9	Inspection after unloading
$C\left(m_{3}:m_{4}\right)$		$C\left(m_{6},m_{15},m_{16}:m_{18}\right)$	
T3	Transportation	T10	Moving to the next stage
$C\left(m_{21}:m_{4},m_{5},m_{6},m_{22}\right)$		$C\left(m_{16}:m_{17}\right)$	
T4	Reading time‐temperature data	T11	Urgent processing
$C\left(m_{5}:m_{7}\right)$		$C\left(m_{18}:m_{19}\right)$	
T5	Examining temperature fluctuations	T12	Launched GPS
$C\left(m_{7},m_{8}:m_{9},m_{13}\right)$		$C\left(m_{20}:m_{21}\right)$	
T6	Risk warning of temperature and launching regulation mechanism	T13	Extracting information from the GPS
$C\left(m_{9}:m_{10}\right)$		$C\left(m_{22}:m_{23}\right)$	
T7	Examining temperature fluctuations	T14	Uploading GPS data to the information communion center
$C\left(m_{10}:m_{11},m_{12},m_{13}\right)$		$C\left(m_{23}:m_{14}\right)$	

**Figure 4:**
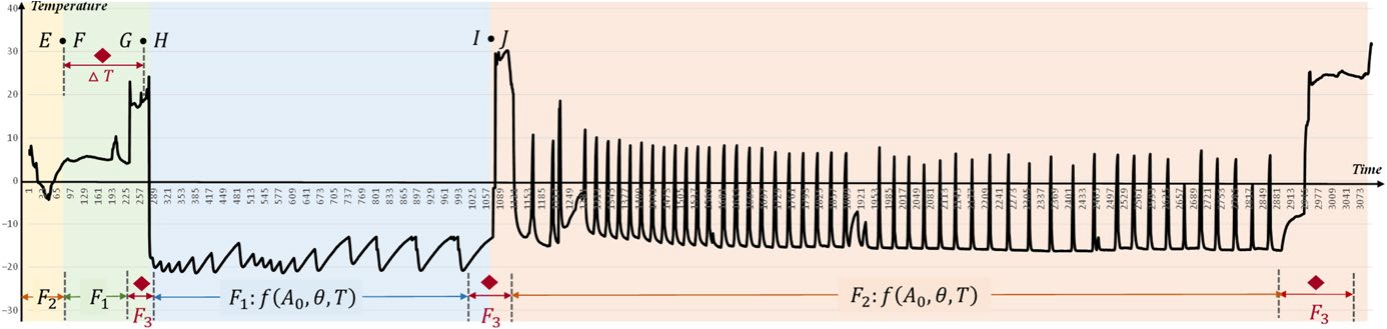
The whole‐process temperature information for the cold chain of tilapia. The analysis of the entire temperature data as the most critical information is shown by extracting temperature attribute value for nodes across the entire cold chain

The process of tilapia in the cold chain is taken as an example to analyze the validity of the modeling methodology which is researched on verification of the quality safety. The analysis of the entire temperature data as the most critical information is shown in Figure 4 by extracting temperature attribute value for nodes across the entire cold chain. According to the multihierarchical network of four‐dimensional graphics, the feature description of temperatures before the tilapia enter the processing stage is used by F2 mathematical expression. The quality assessments at the processing stage and the storage stage of tilapia are analyzed by the formula F1. The quality assessment modeling at the transportation stage of the cold chain is defined by algorithm F2. At each level of convergence, the temperature fluctuations are very violent. Then, the model F3 is used under critical conditions. The red diamond logo is the key node information of quality assessments.

In this research, temperature data is shown in Tables 9, 10, 11 for the temperature fluctuations across every hierarchy of the cold chain.

**Table 9:** EF temperature data under critical conditions

Tem.1	Tem.2	Tem.3	Tem.4	Tem.5	Tem.6	Tem.7	Tem.8	Tem.9	Tem.10
5.44	3	–0.94	–3.44	–3.19	–0.06	2.25	4	4.94	4.63
4.38	2	–1	–3.81	–3	0.25	2.38	4.13	5.06	4.63
3.81	0.13	–1.19	–3.87	–2.31	0.56	2.56	4.25	5.13	4.63
3.56	–0.12	–1.37	–4	–1.37	0.94	2.69	4.44	4.94	4.56
3.5	–0.5	–1.44	–4	–0.94	1.25	2.88	4.56	4.88	4.56
3.81	–0.5	–1.37	–4.06	–0.69	1.44	3.06	4.56	4.81	4.56
3.19	–0.5	–2.56	–3.56	–0.56	1.63	3.31	4.56	4.75	4.56
3.31	–0.56	–2.12	–3.12	–0.44	1.81	3.5	4.69	4.69	4.56
3.94	–0.69	–2.06	–4.37	–0.37	2	3.69	4.81	4.69	4.63
4.13	–0.81	–2.94	–3.62	–0.25	2.13	3.88	4.88	4.63	4.63

**Table 10:** GH temperature data under critical conditions

Tem.1	Tem.2	Tem.3	Tem.4	Tem.5	Tem.6	Tem.7	Tem.8	Tem.9	Tem.10
5.63	5.44	5.06	4.81	8.75	4.94	4.06	17.94	17.63	20.75
5.63	5.38	5.06	4.88	8.94	4.88	4.06	18	17.81	21.25
5.63	5.38	5.06	4.88	8.94	4.81	4.13	18	20.44	19.63
5.63	5.38	5	4.88	9.94	4.69	4.13	17.88	18.63	19.13
5.63	5.31	5	4.88	10.31	4.63	4.19	17.63	18.31	18.88
5.63	5.31	5	4.94	9	4.56	4.25	17.44	18.31	24.13
5.63	5.25	5	4.94	9	4.5	23	17.38	18.38	–13
5.56	5.25	4.94	5	7.5	4.44	20.38	17.13	18.44	–16.69
5.56	5.25	4.94	5	6.63	4.38	17.5	17.13	18.69	–17.63
5.56	5.19	4.94	5	6.13	4.38	17.5	17	18.75	20.75
5.5	5.19	4.88	5.94	5.88	4.31	17.63	17.13	18.88	21.25
5.5	5.13	4.88	7.44	5.63	4.13	17.75	17.13	18.88	19.63
5.5	5.13	4.88	7.38	5.38	4.13	17.69	17.25	19.13	19.13
5.44	5.13	4.88	7.81	5.25	4.06	17.81	17.25	19.19	18.88
5.44	5.13	4.88	8.5	5.06	4.06	17.88	17.56	19.25	24.13

**Table 11:** IJ temperature data under critical conditions

Tem.1	Tem.2	Tem.3	Tem.4	Tem.5	Tem.6	Tem.7	Tem.8	Tem.9	Tem.10
−17.25	−13.69	−11.31	−9.38	−7.75	−6.31	−5.13	−4.13	−3.25	−2.5
−17.13	−13.56	−11.25	−9.31	−7.69	−6.25	−5.13	−4.06	−3.19	−2.5
−16.94	−13.5	−11.19	−9.25	−7.63	−6.25	−5.06	−4.06	−3.19	−2.5
–16.81	–13.38	–11.13	–9.19	–7.63	–6.19	–5	–4	–3.13	–2.44
–16.69	–13.31	–11.06	–9.19	–7.56	–6.13	–5	–3.94	–3.13	–2.44
–16.56	–13.19	–11	–9.13	–7.5	–6.13	–4.94	–3.94	–3.13	–2.38
–16.44	–13.13	–10.94	–9.06	–7.44	–6.06	–4.94	–3.88	–3.06	–2.38
–16.31	–13.06	–10.81	–9	–7.44	–6	–4.88	–3.88	–3.06	–2.38
–16.19	–13	–10.75	–8.94	–7.31	–6	–4.81	–3.81	–3	–2.31
–16.06	–12.88	–10.69	–8.88	–7.31	–5.94	–4.81	–3.81	–3	–2.31
–15.94	–12.81	–10.63	–8.81	–7.25	–5.88	–4.75	–3.81	–3	–2.31
–15.81	–12.75	–10.56	–8.75	–7.19	–5.88	–4.75	–3.75	–2.94	–2.31
–15.69	–12.69	–10.5	–8.69	–7.19	–5.81	–4.69	–3.69	–2.94	–2.25
–15.63	–12.56	–10.44	–8.63	–7.13	–5.75	–4.69	–3.69	–2.88	–2.25
–15.5	–12.5	–10.38	–8.56	–7.06	–5.75	–4.63	–3.69	–2.88	–2.25
–15.38	–12.44	–10.31	–8.5	–7	–5.69	–4.63	–3.63	–2.81	–2.19
–15.25	–12.31	–10.25	–8.44	–7	–5.69	–4.56	–3.63	–2.81	–2.19
–15.13	–12.25	–10.19	–8.44	–6.94	–5.63	–4.5	–3.56	–2.81	–2.19
–15	–12.19	–10.13	–8.38	–6.88	–5.56	–4.5	–3.56	–2.75	–2.19
–14.88	–12.13	–10.06	–8.31	–6.81	–5.56	–4.44	–3.5	–2.75	–2.13
–14.75	–12	–10	–8.25	–6.75	–5.5	–4.44	–3.5	–2.75	–2.13
–14.63	–11.94	–9.94	–8.19	–6.75	–5.44	–4.38	–3.5	–2.69	–2.13
–14.5	–11.88	–9.88	–8.19	–6.69	–5.44	–4.38	–3.44	–2.69	–2.06
–14.44	–11.81	–9.81	–8.13	–6.63	–5.38	–4.31	–3.44	–2.69	–2.06
–14.31	–11.75	–9.75	–8.06	–6.63	–5.31	–4.31	–3.38	–2.63	–2.06
–14.19	–11.69	–9.69	–8	–6.56	–5.31	–4.25	–3.38	–2.63	–2
–14.13	–11.63	–9.63	–7.94	–6.5	–5.25	–4.25	–3.31	–2.63	–2
–14	–11.5	–9.56	–7.88	–6.44	–5.25	–4.19	–3.31	–2.56	–2
–13.88	–11.44	–9.5	–7.88	–6.44	–5.19	–4.19	–3.25	–2.56	–1.94
–13.75	–11.38	–9.44	–7.81	–6.38	–5.19	–4.13	–3.25	–2.5	–1.94

Based on the above model, operating safety accidents generally occur at the processing stage. Time span of processing tilapia is a significant parameter to indicate the operating safety which is evaluated by extracting attributes notes of time from m_1_ to m_26_ in Table 4. The demand on limit throughput time via processing can be solved to study the measurement of aquatic products. The limited time span of fresh aquatic products is 40 min according to HACCP (Hazard Analysis and Critical Control Point). Attribute values of the beginning nodes and the ending nodes in the processing stage are extracted as time data from a set of multidimensional information in the network‐hierarchy modeling. That modeling analyzes the processing time data to estimate the proper time span for keeping tilapia fresh. The simulation experiment is executed in the cold chain of tilapia. A batch of thirty tilapia (500–650 g) are captured from the pond in Haikou of China. The business‐logic codes by attributes extraction of multidimensional data are shown as follows:

$$\begin{array}{c} \text{If}\;\;\;\theta \;\left(m_{25}\right)-\theta \;\left(m_{1}\right)\leq 40\\ \text{then}\;\{M\left(m_{26}\right)=\text{true},\\ i=M(m_{26})+1,\\ \text{Enabled}\;(m_{i})=\text{true}\};\\ \text{Else}\;\{M\left(m_{26}\right)=\text{false},\\ i=M(m_{26})+1,\\ \text{Enabled}\;(m_{i})=\text{false}\}. \end{array}$$

The processing is considered to be safe enough to trigger the next events within 40 min only if the decisive variable quantity Enabled (*m*_*i*_) is “true.” Providing that Enabled (*m*_*i*_) is false, the quality analysis of aquatic products has to be implemented instead of triggering the next events; then, the processing time is over 40 min. Time–temperature data in the processing stage is revealed. The highest temperature for tilapia is 7.8°C while the lowest is 2.4°C at the processing stage in the cold chain. Time–temperature data is acquired from sensors every 30–s as it is estimated. There are 174 pieces of data records. The processing time within 29 min is approved to be safe according to the decision‐making modeling of quality assessments in the cold chain.

Periodical quality calculation is acquired based on time‐temperature variable quantity which is the parameter in the quality assessment modeling. Therefore, quality variation of aquatic products is analyzed and evaluated during the circulation of the cold chain.

In the experiment, time‐temperature data of tilapia is aggregated in the processing stage, the storage stage, and the transportation stage during 12.33 days (17,753 min). Environmental temperature equipped with the Labuza modeling is practicing on quality assessments and shelf life estimations frequently. The related values of quality assessments become dimensionless parameters, while the quality decay occurs eventually.

Quality control and shelf life estimations mostly depend on the time ranging from the processing stage, the storage stage to the consumption stage. Temperature control in the primary period of processing and transportation is extremely important for the quality safety of aquatic products in the cold chain. Thus, temperature fluctuations take an incredible impact on product quality of tilapia, especially in the processing stage and the transportation stage as the intensive and violent temperature fluctuations occur. Temperature in the storage stage is almost stable, although temperature in the transportation stage is fluctuated massively. Experimental data is calculated through TTT protocol and shelf life estimation modeling to verify enhancement of decay ratio of tilapia as time goes. Apparently, continuous temperature reduction is launched as decision‐making response strategies. Mathematically, the quality of tilapia is declined along with changes of environmental temperatures during the cold chain. The quality of tilapia is separated into five intervals, in which better quality interval range is 0.8–1 with relevant time 6 days and worse quality interval range is 0–0.2 with relevant time 24 days. The forth interval (0.2–0.4) is chosen to be a reference value of safe quality of tilapia with expiration date 18 days according to the quality assessment modeling. More specifically, expiration date 18 days is the outcome of quality decay estimation based on the Labuza modeling with the experimental time‐temperature data. In the extensive experiment of tilapia, expiration date 17.13 days almost equals the 18 days, which is calculated based on necessary data, such as actual circulating time 12.33 days in the cold chain and expiration date 4.8 days under the 1.5°C environmental temperature.

## 6 CONCLUSIONS

In this research, multidimensional information is collected to describe transmission information of the cold chain. It is a solution to aggregate analysis of information management with inadequate environmental information and quality information. The hierarchical network modeling is established through the analysis of transmission information for full‐dimensional information collections and seamless information records. A diversified and profound information collection and information transmission of the entire cold chain is conducted by multidimensional information in the network modeling. The wide and complete information description and information modeling are created based on the information transmission in the cold chain. Information of tilapia is studied intensively with the multidimensional information consisting of the processing stage, the storage stage, and the transportation stage from the hierarchical network modeling. The information is recorded and transmitted in the entire cold chain to compose the behavior information clustering, the environmental information clustering, and the quality information clustering, respectively. Then, operating safety and quality assessments are analyzed and evaluated by the methodology. Conclusively, the network‐hierarchy modeling with multidimensional information tends to conclude comprehensive analysis of environmental information and quality information, which ensures that the quality of aquatic products is evaluated precisely and accurately during the entire circulation of the cold chain. This model is available in effective information managements of aquatic products to provide significant references for decision‐making strategies which guarantee the safety of the cold chain. From the aspect of aquatic product management, information integration and in‐depth analysis of the whole process of cold‐chain aquatic products can be performed from the aspect of multidimensional information using this model from a single operation or independent temperature record. The overall management level of aquatic products can be improved, and risks of aquatic products can be managed and identified with early warnings. Thus, the safety of aquatic product cold chain can be enhanced.

## CONFLICT OF INTEREST

The authors declare that they do not have any conflict of interest.

## ETHICAL REVIEW

This study did not involve any human or animal testing. This study was approved by the Institutional Review Board of Ludong University.

## INFORMED CONSENT

Written informed consent was obtained from all study participants.
